# Clinical Group Students/Trainees Essay Contest Winner “A Rising Leader: Bangladesh”

**DOI:** 10.4269/ajtmh.25-0546

**Published:** 2026-02-10

**Authors:** Rizwana Khan, Md. Khalequzzaman

**Affiliations:** Enteric and Respiratory Infections, Infectious Disease Division, International Centre for Diarrhoeal Disease Research, Bangladesh, Dhaka, Bangladesh

## Abstract

The American Committee on Clinical Tropical Medicine and Travelers’ Health’s Students/Trainees Leadership Group launched their essay contest in 2025 to encourage the involvement of emerging voices in tropical medicine in key issues shaping the field’s present and future.

The following authors’ won for their essay addressing the following prompt:

“*The Role of International Cooperation in Combating Tropical Diseases*”

These essays were reviewed by a panel of clinician judges with experience in tropical medicine and writing, and the winning entries reflect both academic excellence and practical insight. Together, the two winning essays show the wide scope of tropical medicine from policy and prevention to diagnosis and treatment and the shared responsibility to confront these challenges wherever they arise.

## BACKGROUND

In the dense humidity of a Bangladeshi summer, I once watched a young mother cradle her febrile child outside a rural clinic. Her child had dengue. There was no intensive care unit within a hundred miles, no rapid diagnostics, and no certainty that she would survive the night. Across the globe, similar scenes unfold every day in communities burdened by tropical diseases that thrive where infrastructure falters and inequities deepen. Behind the suffering is an undeniable truth: tropical diseases respect no borders, and neither should the fight against them. Microbes are cosmopolitan and combating them demands unwavering international cooperation.

## FACING CHALLENGES, FINDING HOPE

Bangladesh’s epidemiological profile reflects a perfect storm of conditions conducive to tropical disease proliferation: a tropical monsoon climate, rise in temperature, rapid urbanization, limited and basic sanitation in periurban slums, and periodic climate-induced disasters like floods and cyclones. These factors amplify transmission of vector-borne diseases like dengue and waterborne illnesses such as cholera.

Bangladesh is a country of innovation and hope. The International Centre for Diarrheal Disease Research, Bangladesh (icddr,b) exemplifies how global partnerships can yield transformative local impact. From pioneering oral rehydration therapy to hosting large-scale vaccine trials, icddr,b’s success is a testament to what’s possible when international resources align with national priorities.

## DENGUE: A BORDER LESS CRISIS

The dengue epidemic in Bangladesh underscores the transnational nature of tropical diseases. In 2023, the country reported 321,017 cases admitted to hospitals and more than 1,600 deaths, the highest on record. The *Aedes aegypti* mosquito, the vector for dengue, recognizes no borders, nor do the climate patterns and urban migration trends that fuel its proliferation.

Addressing this crisis demands regional entomological research collaborations, crossborder vector monitoring, and coordinated policy action across South Asia. Existing mechanisms such as the South Asian Association for Regional Cooperation health framework and WHO’s South-East Asia Regional Office offer platforms for this, but they remain underutilized. Greater integration, funding for vaccine development, and community-based vector control are critical next steps.

## CHOLERA: A TEST CASE FOR GLOBAL HEALTH EQUITY

Cholera, long endemic to Bangladesh, remains a litmus test for international health cooperation. Climate change, specifically changing rainfall, and temperature outbreaks of diarrheal diseases, including cholera is re-emerging. In 2022 icddr,b Dhaka hospital reported over 1,200 cases in a single day. The oral cholera vaccine, developed with support from the International Vaccine Institute and produced by South Korean and Indian manufacturers, was piloted in slums of Dhaka city through a remarkable collaboration involving the Ministry of Health, icddr,b, and Gavi.

However, global stockpiles remain insufficient, and international allocation decisions can leave highly vulnerable populations in countries like Bangladesh underserved. This is where cooperation must evolve into equity. Technology transfer and local vaccine manufacturing are critical to sustainable control of cholera. In addition to this, improving water, sanitation and hygiene can play an important role in combating cholera.

## BUILDING RESILIENCE THROUGH SHARED SURVEILLANCE

Tropical diseases often spread unnoticed when outbreaks aren’t reported, data isn’t shared, and research remains isolated. Real-time, crossborder disease surveillance offers a powerful solution. Lessons from the coronavirus disease 2019 pandemic have strengthened digital tools and genomic sequencing capacity that can now be applied to neglected tropical diseases. Bangladesh’s engagement in the Global Health Security Agenda is promising, but real progress depends on stronger data-sharing, trust, and coordinated regional action.

## THE CLIMATE NEXUS: NO COUNTRY CAN STAND ALONE

Climate change is altering disease ecology. Rising temperatures, erratic rainfall, and salinity intrusion are shifting the geographic distribution of vectors and pathogens. Bangladesh, with its deltaic vulnerability, is on the front line of this crisis. Predictive modeling, artificial intelligence-powered outbreak forecasting, and climate-resilient health care systems are global goods. They require pooled investment and expertise, not isolation.

International partnerships must not only respond to outbreaks but anticipate them by investing in climate-disease modeling hubs, regional biosurveillance, and joint simulation exercises. This must become the norm, not the exception.

## FROM AID TO ALLIANCE: RETHINKING COOPERATION

International cooperation must mature beyond donor-recipient dynamics. Bangladesh with its strong public health workforce, clinical trial infrastructure, and experience in vaccine campaigns can lead south–south cooperation. Moreover, research partnerships must shift toward equity-centered collaboration. Local scientists must have authorship rights, decision-making power, and access to global platforms.

## CONCLUSION: A VISION FOR SHARED VICTORY

Combating tropical diseases in Bangladesh is not merely a public health imperative it is a matter of global security and justice. The microbes do not care for borders, and neither should our response. International cooperation is not a diplomatic ideal, it is an operational necessity.

Bangladesh’s experience shows that when international support aligns with local leadership, the results can be extraordinary.

The path forward lies in deeper partnerships, mutual respect, and a shared vision where a child in rural Rajshahi has the same right to health as one in New York or Geneva.

The future of tropical disease control does not lie in isolated victories but in shared resilience. Let Bangladesh be not only a recipient of global solidarity but a model of how it can be built and how, together, we can defeat diseases that have plagued us for centuries.

